# Molecular Plasticity of the Nucleus Accumbens Revisited—Astrocytic Waves Shall Rise

**DOI:** 10.1007/s12035-019-1641-z

**Published:** 2019-05-27

**Authors:** Julianna Kardos, Árpád Dobolyi, Zsolt Szabó, Ágnes Simon, Guillaume Lourmet, Miklós Palkovits, László Héja

**Affiliations:** 1 Functional Pharmacology Research Group, Institute of Organic Chemistry, Research Centre for Natural Sciences, Hungarian Academy of Sciences, Magyar tudósok körútja 2, Budapest, 1117 Hungary; 2 Laboratory of Neuromorphology, Department of Anatomy, Histology and Embryology, Semmelweis University, Üllői út 26, Budapest, 1086 Hungary; 3 MTA-ELTE Laboratory of Molecular and Systems Neurobiology, Department of Physiology and Neurobiology, Eötvös Loránd University and the Hungarian Academy of Sciences, Pázmány Péter sétány 1C, Budapest, 1117 Hungary; 4 Human Brain Tissue Bank, Semmelweis University, Tűzoltó utca 58, Budapest, H-1094 Hungary

**Keywords:** Nucleus accumbens macrosystem, Motivation-reward metaplasticity, Mixed GABAergic and Gluergic synapses, Perisynaptic astrocytic processes, Astrocytic endfeets, Succinate receptor

## Abstract

Part of the ventral striatal division, the nucleus accumbens (NAc) drives the circuit activity of an entire macrosystem about reward like a “flagship,” signaling and leading diverse conducts. Accordingly, NAc neurons feature complex inhibitory phenotypes that assemble to process circuit inputs and generate outputs by exploiting specific arrays of opposite and/or parallel neurotransmitters, neuromodulatory peptides. The resulting complex combinations enable versatile yet specific forms of accumbal circuit plasticity, including maladaptive behaviors. Although reward signaling and behavior are elaborately linked to neuronal circuit activities, it is plausible to propose whether these neuronal ensembles and synaptic islands can be directly controlled by astrocytes, a powerful modulator of neuronal activity. Pioneering studies showed that astrocytes in the NAc sense citrate cycle metabolites and/or ATP and may induce recurrent activation. We argue that the astrocytic calcium, GABA, and Glu signaling and altered sodium and chloride dynamics fundamentally shape metaplasticity by providing active regulatory roles in the synapse- and network-level flexibility of the NAc.

## Organization of the Nucleus Accumbens

Nucleus accumbens (NAc) is part of the ventral striatal division where circuit afferents and efferents both unite and segregate [61, 136] in distinctive neuronal ensembles [154]. Discernible NAc sub-territories of rodents, the “chameleon-like” shell and the core [67, 83, 186, 237, 241], are associated with the limbic and the motor systems, respectively [215]. In addition, core and shell sub-regions have many more functions, including incentive-cue responding and behavioral inhibition (see for example [6]). While rodent shell and core sub-regions and related neuronal circuit connections are clearly distinguishable [237], sub-region borders of human NAc are less apparent, displaying more diffuse, gradual changes in the topology of afferents and efferents [52, 107, 137]. We suggest that the characteristic differences between rodent and human NAc sub-territories are related to the diverse incentive-cue responding and behavioral inhibition of humans.

The major neuronal type in the nucleus accumbens is the medium spiny neuron (MSN), which comprise about 95% of the cells in the area. Neurochemical phenotypes of MSNs range from “quasi” inhibitory using the major inhibitory neurotransmitter γ-aminobutyric acid (GABA) to mixed inhibitory and excitatory (GABAergic and glutamatergic). Besides, ubiquitous distribution of terminals co-expressing vesicular glutamate (Glu) and GABA transporters in the striatum, hippocampus, thalamus, and cerebellar and cerebral cortices [45] suggests that the appearance of mixed Glu-GABA phenotypes may possibly be the rule rather than the exception (for a more thorough discussion on the possible significance of the mixed Glu-GABA MSN phenotype in the NAc, see the last paragraph of section “Basic Neurochemistry of Reward Quality and Prediction”). Accumbal MSNs exhibiting both GABA and Glu decarboxylase (GAD) immunoreactivity [5, 7, 227, 238] often co-express modulatory neuropeptides (substance P, dynorphin, enkephalin, and neurotensin) together with various dopamine (DA) receptor subtypes (DR1, DR2, and DR3). The DR1-DR2 receptor heteromer-expressing phenotype also takes up [^3^H]aspartate ([^3^H]Asp) [156, 227]. The major DAergic input driving the different DA receptor types originates in the ventral tegmental area (VTA), while Gluergic inputs to the NAc arrive mostly from cortical areas. The latter innervations, however, also terminate on MSNs, raising the idea of “striatal synaptic triad.” This represents a configuration of a Gluergic asymmetric spine head with a DAergic symmetric spine neck [50, 62, 188], although asymmetrical morphology has also been considered [16, 100, 228, 239].

Interneurons (< 5%) in the NAc are mainly GABAergic, and to a lesser extent cholinergic, receiving serotonergic inputs [192, 218, 238] in both the shell and core regions. The GABAergic interneurons exhibit nitric oxide synthase activity and somatostatin (SOM) and neuropeptide Y or parvalbumin (PV) expression. Gluergic input to the accumbal SOM expressing interneurons [169] may possibly evoke release of SOM specifically signaling to astrocytes [122]. The PV-expressing sub-population of interneurons has recently been noted as a major player in amphetamine sensitization and reward [226]. Also, we conjecture that the GABAergic PV-expressing NAc interneurons control the fast-firing MSNs, thereby shaping accumbal sensitization (for explanation and references cf. the last paragraph of the “Unique Glu-GABA Drives of the NAc” section). The GABAergic interneurons also receive both DAergic input from the VTA and glutamatergic innervation from cortical areas and in turn terminate on MSNs. Recently, Gluergic input from the VTA terminating on both interneurons and MSNs has also been established. This is the only Gluergic input to the NAc, which mediates aversion instead of reward [163]. Another small proportion of NAc neurons are tonically active cholinergic interneurons, which are the only source of acetylcholine (Ach) in the NAc [112]. These cells receive mostly Gluergic but also ascending serotonergic inputs and synapse onto MSNs through nicotinic (nAChR) and muscarinic acetylcholine (mAChR) receptors, which exert opposing effects on DA signaling. Whereas nAChR activation diminishes, mAChR activation increases motivation toward reward-predicting cues ([38, 39] These cells were also identified as central players in the development of depression-like symptoms, because the disruption of cell surface expression of serotonin (5HT) receptors and/or other ion channels on cholinergic interneurons had antidepressant actions with therapeutic potential [225]. As to the molecular mechanisms, the expression and function of the hyperpolarization-activated cyclic nucleotide-gated channel 2 was suggested to be important as its overexpression in cholinergic interneurons was sufficient to rescue depressive phenotypes [31]. Recently, activation of serotonergic innervation from dorsal raphe nucleus to NAc was also found to be a prerequisite for normal social interaction in mice [222]. These findings qualify petite assemblies of accumbal interneurons as governing big networks associated with behavioral regimes. The operational blowup of interneuron activities shall require local and long-range neuro-glia coupling, to keep pace with the extreme energy demand of real-time dynamics of various molecular players and with the remodeling of synaptic morphology and neuronal circuitries.

## Basic Neurochemistry of Reward Quality and Prediction

Reward sensitivity critically depends on the DA neurotransmitter system [19, 47, 203]. Incoming DAergic activity from the VTA in the NAc not only affects activity of the neuronal network but also affects astrocytic calcium signals, since they are dynamically modulated by D2R receptor activation [234]. In addition, DAergic stimuli induce the synthesis of modulatory neuropeptides, like dynorphin, enkephalin, neurokinin A/B, neurotensin, and substance P in astrocytes. The action mechanisms of neuropeptides in the NAc are particularly interesting within the framework of the future development of psychiatric drugs [59]. The DAergic VTA input in the NAc can regulate DA level by feedback mechanisms using collaterals to midbrain DA neuron areas. The incoming VTA signal affects neurons in the rostrodorsal and caudal parts of NAc differently (cf. “hotspots” and “coldspots” referenced below) based on separate co-expression patterns of various DA and opiate receptor subtypes. Endogenous ligands of opioid receptors, enkephalins, modulate locomotor activity by the facilitation of presynaptic DA release. D1R-positive MSNs express mu-opioid receptors predominantly, whereas D2R-positive neurons respond to delta and kappa ligands [7, 29]. Mu-opioid receptor agonists induce not only food intake but also food-reinforced operant behavior [185]. In contrast, accumbal DA receptor activation with amphetamine does not bear any feeding motivation component ([198], but see [194, 217]); nevertheless, it still produces self-stimulation behavior [23]. Opioids/cannabinoids or anandamide evoke their gustatory hedonic reaction by activating receptors distributed in a well-defined anatomical pattern, in the so-called “hotspots” in the NAc shell [29, 78, 102, 119, 148]. Together with mu-opioid receptors, delta- and kappa-opioid receptors are also clustered in the rostrodorsal region of NAc, enhancing gustatory hedonic reaction (“liking”). In contrast, the very same receptors mediating hedonic suppression map to the caudal part of NAc (“negative hedonic coldspot” [29, 30]).

Accumbal instrumental learning [13, 27, 40, 64, 72, 91, 145, 178, 195, 235] is a fundamental capability of an animal to weigh the utility of selected actions against the expected outcomes. This concept involves occurrence-dependent strengthening of response open to different interpretations—that is, putting either “interaction” [171] or “reward” [18, 19, 180] aspects in the limelight. In this respect, NAc is considered to be the main hub of the brain that—depending on the actual status of the ascending inputs from limbic structures—exercises sharp bivalent control over the operant behavioral output. Various types of in vivo NAc stimulation paradigms consistently yield opposite animal behavior: either reward/appetitive or stress/aversive. The receptive fields of afferent fibers from prefrontal, entorhinal cortex, amygdala, or hippocampus show little spatial overlap, but individual NAc projecting neurons (GABAergic MSNs) demonstrate a high degree of synaptic convergence from the same input regions [142, 143]. MSNs with mixed GABA-Glu phenotypes [156, 227] could well serve this principle. It is conceivable that at mixed GABA-Glu synapses, the ratio of Glu over GABA co-released from these cells depends on the strengths and frequency of varied input stimulations [44, 141, 189]. Activity-dependent shifting of the balance between GABA and glutamate release allows fine-tuning of transmission probability via changing prevalence of the inhibitory component (GABA). This way, accumbal MSNs with mixed GABA-Glu phenotypes predispose NAc to signal and drive positive or negative conducts.

## Unique Glu-GABA Drives of the NAc

The medial prefrontal cortex relays taste information from the primary insular cortex, which constitutes the neuronal basis of food intake and energy homeostasis [20]. Local inhibition of ionotropic Glu receptors (or activation of GABA_A_ receptors) in the shell region of NAc evokes strong feeding response (or positive place preference in other experimental paradigms) by inhibiting MSNs that disinhibit upstream targets like the lateral hypothalamus, ventral pallidum (VP), or VTA [198]. Early studies indicated that the major excitatory input from the medial prefrontal cortex to the anterior pole of NAc (cortico-accumbal pathway) uses Glu or Asp as neurotransmitter [36, 37]. Subsequently, it was demonstrated that feeding induces ambient (Glu) increase in the lateral hypothalamus and decrease in the accumbal (Glu) that was detected by microdialysis probes inserted into the NAc [164]. NAc receives Gluergic inputs from the ventral hippocampus [12, 21, 95] suggesting that depression and drug/ethanol reward behaviors are furthered via the strengthening of these synapses. Recently, a chemogenetic approach has been applied to distinguish the contribution of the activation of VTA-GABA neurons from other mesoaccumbal nerve terminals to incentive salience. The results indicate that VTA-GABA neurons, but not GABA projections, disrupt incentive salience processes [221].

Several lines of evidence support the crucial aspects of NAc in drug reward modulation [10, 60, 103, 157, 243]. Upon chronic exposure to cocaine, the accumbal alpha-amino-3-hydroxy-5-methyl-4-isoxazole propionate receptor (GluA2/AMPA receptor) is upregulated [92], and NMDA receptor–dependent long-term depression in MSNs in the core region of NAc is suppressed [88]. It is to note that extinction and reconsolidation of cocaine seeking behavior monitored by mass spectrometry–based phosphoproteomics disclosed Gluergic basolateral amygdala inputs to NAc as being crucial for cocaine cue exposure [209]. The drug-seeking behavior could be associated with synaptic changes, such as dendritic spine head diameter and AMPA/NMDA receptor ratio [197]. Extracellular Glu in the NAc is modulated by group 2 metabotropic Glu receptors [233]. Group 2 and 3 metabotropic Glu receptors operate at prefrontal cortical axon terminals and modulate DAergic transmission at the same synapse [121]. Although Glu or GABA activation can evoke similar positive/negative motivational patterns, the effect of GABA holds a hedonic component as well. The major source of GABAergic innervation in the NAc arises from local aspiny interneurons [7, 15]. Apparently, these neurons provide the feed-forward inhibition of neighboring MSNs during excitatory stimulation from descending cortical and hippocampal structures. Presynaptic GluK1/2 heterodimer kainate receptors at cortical afferents play a major role in this inhibition of MSN activity, because GluK1/2 receptor activation decreases glutamatergic but increases GABAergic synaptic transmission in the NAc [28, 39].

The “all or none” type of control of fast-spiking MSNs by the GABAergic PV-containing accumbal interneuron ensemble implies unique functional significance [101, 104, 238] such as sensitization [208]. The bivalent nature of NAc output [167, 168, 170] to different basal ganglia and mesencephalic structures is discernible already at the electrophysiological characteristics of the MSNs that also show bistability [105]. MSNs display spontaneous transition of membrane potential between a more hyperpolarized, resting “down state” and a more depolarized, active “up state”—only when barrages of action potentials can be discharged [142]. Similarly, the influence of hippocampal interneurons on the output of cooperating principal cells would serve to induce synaptic enhancement in target structures during behavioral inactivity, consumer behaviors, and slow-wave sleep [25]. Based on findings showing that cortical astrocytes play an indispensable role in cortical state switching [162] and even in the generation of genuine, physiological slow-wave activity in vivo [200], it is suggested that astrocytes may trigger the same coordination of neuronal “up” and “down” state oscillations of accumbal MSNs. Consequently, the heavily gap junction–coupled, easily synchronizable astrocyte network may significantly contribute to the coordinated activation of the NAc circuitry, eventually establishing synaptic reinforcement (see also “Rising Astrocyte Waves: New Layers of Accumbal Neuro-Glia Coupling” section).

## Modulation of Inhibitory Signaling by Converging Metabolic and Reward Pathways

Emerging themes, like cellular stress, hypoxia, and inflammation, are examples of functional association between signaling molecules and citrate energy cycle (CEC) metabolites [211], primarily succinate (Sucn) [34, 124, 158, 202]. Fumarate accumulation associated with glutaminolysis also presents a hallmark of cellular defense mechanism [9]. Mutations of the mitochondrial succinic semialdehyde gene (aldo-keto reductase Aldh5A1) cause succinic semialdehyde dehydrogenase (SSADH) deficiency [120, 219]. In this case, the conversion of SSA to Sucn by SSADH is diminished, while the accumulation of γ-hydroxybutyric acid (GHB) from GABA is maintained. Different responses to methadone maintenance treatment have been explained by a deviation of GABA catabolism from the CEC due to altered Aldh5A1 expression in opioid-dependent patients [48].

Genes repressed in the NAc and the frontal cortex (FC) of cocaine-, morphine-, and ethanol-vulnerable Lewis rats [73] help to uncover associated signaling and metabolism, underlying the manifestation of addiction, an important behavioral extremity. Higuera-Matas and co-workers [73] highlighted some genes as being associated with (i) changes in the striatum of cocaine-sensitized rats (parvalbumin/Pvalb) [208]; (ii) drug addiction (sphingomyelin synthase, Sgms2) [177]; and (iii) methamphetamine (Meth)–induced psychosis (NAD(P)H dehydrogenase, Nqo2) [79, 146]. Importantly, genes for Sucn receptor 1 (Sucnr1) and aldo-keto reductase AKR1B10 (Akr1b10) involved in Sucn biosynthesis were also repressed in both the FC and the NAc of Lewis rats [73]. Indeed, the significant role for PV-positive accumbal interneurons in drug-related learning is substantiated by recent data demonstrating PV-positive GABAergic interneurons as a prerequisite for psychostimulant (amphetamine)–induced behavioral adaptation [[223]; discussed by [226]].

Expression data alone may not be sufficient to explain changes in network stability or infer causality in reward/addiction process. Yet, these considerations and findings may allow speculations on a possible functional link between the danger signal Sucn [202] and reward/addiction processes. Both the coincidence and synergy of ATP- and Sucn/GHB-responsive astrocytic calcium transients together with the presence of high-affinity Sucn/GHB recognizing sites in the NAc [128–133] strongly imply the involvement of a Sucn-responsive astrocytic target. This positive feedback mechanism supports the nucleation-type model [118] of the astrocyte network activation dynamics in the NAc. The “Sucn signal” could directly target some GPR91-type G protein–coupled Sucn receptors [8, 65, 131, 166, 175, 179, 210]. The expression of P2Y1 receptors in accumbal astrocytes [49] and P2Y1 receptor antagonist sensitivity of the Sucn- and ATP-responsive calcium transients sensibly predicts interference of Sucn and P2Y1 receptor–mediated calcium mobilization [131]. Nevertheless, the potential validation of brain-type Sucn receptors may reveal distinguishable binding crevices (Table 1) [242]. Sucn binding could also be linked to the mitochondrial Sucn dehydrogenase for which Sucn is the substrate [84, 199, 201]. Also, Sucn may function through some mitochondrial dicarboxylate carriers (Table 1) [80, 139, 150]. It is worth mentioning that neuronal activity–independent calcium bursts have been described in the Bergmann glia of behaving animals and were found to be purinergic [140]. It is conceivable that coincidence of both Sucn- and ATP-responsive calcium bursts at the blood-brain barrier (BBB) [131] may in turn reflect neuron-independent activation of Sucn-deficient astrocytes by the micromolar Sucn supply from blood [65].

**Table 1:**
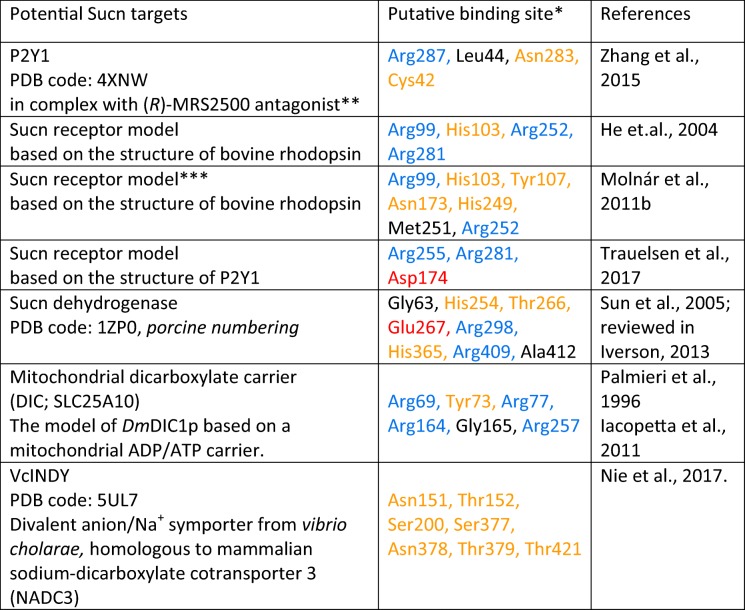
Comparison of putative binding sites of potential Sucn targets in the brain

*Basic, acidic, polar, or neutral characters of amino acid residues are indicated with blue, red, yellow, or black colors, respectively

**P2Y1 receptor couples via Gq proteins and stimulates phospholipase C followed by increases in IP3 and mobilization of calcium from intracellular stores [220]

***Amino acids surrounding Sucn within 4 Å

## Rising Astrocyte Waves: New Layers of Accumbal Neuro-Glia Coupling

Maintenance of the significant energy demand of balanced Glu-GABA signaling depends on proper neuro-glia metabolic coupling in various physiological and disease conditions [14, 68, 69, 89, 129, 131, 149, 174, 200, 213, 231]; for reviews see [4, 70, 86, 87, 96, 97, 110, 184, 216]. This dependency is highlighted by the observation that complexes between the astrocytic Glu transporter EAAT2 and the α2 isoform of Na^+^/K^+^-ATPase are concentrated in the perisynaptic astrocytic processes (PAPs), which also indicates a unique role for Glu homeostasis [123]. Thus, we coin the term tripartite metaplasticity that signifies not only the prior record of the synaptic activity of the neuronal (see for example [207] and reference herein) but also that of the astrocytic moduls within the synapse, whereby a new level of “plasticity of synaptic plasticity” (metaplasticity [1, 2]) is attained. Accordingly, we suggest astrocytic activation [3] and tripartite metaplasticity [2, 33, 56, 110], 2011, 2013; [125, 193] as new substrates of behavioral motivation to action driven by the NAc.

Reactive astrogliosis associated with elevated SSA reductase AKR7A2 [159] may serve as a mechanistic clue for the early appearance of both astroglyopathy in cortico-basal degeneration [114] and modulation of reward/addiction behavior [24]. For example, chronic drug abuse is characterized by astrocytic hypertrophy, astrocytopathy, and astrogliosis [53, 94]. These morphological and pathological changes trigger Glu uptake via EAAT2. The ensuing alteration of Glu and GABA homeostasis and pertinent metabolism [11, 181] cause altered glial fibrillary acidic protein (GFAP) [54, 183] and EAAT2 expressions [187].

Regarding the astrocytic control over GABAergic actions, tonic inhibition of the extrasynaptic δ-containing GABA_A_ receptors can be induced by GABA release through the astrocytic GABA transporter (GAT3) due to EAAT2 activation. Moreover, the neuronal activity-dependent exchange of GABA for Glu also influences the power of in vivo gamma oscillations as monitored in the rat hippocampus [69]. This mechanism is adjusted by astrocytic GABA production from polyamines by monoamine oxidize B [69, 236]. Several lines of pharmacological evidence suggest that turning excitation into inhibition by astrocytes may also be relevant to NAc. Reportedly, chronic monoamine oxidase B inhibitor treatment diminished cocaine reward in mice [74]. Also, extrasynaptic δ-containing GABA_A_ receptors in the NAc dorsomedial shell played a role in alcohol intake [138]. It is proposed therefore that the astrocytic Glu-GABA exchange mechanism revealed in the hippocampal formation and the striatum [68, 69, 231]; for reviews see [86, 87, 93, 96, 97, 216] may also modulate NAc functions by adapting tonic inhibition. It is tempting to speculate about the likely correlation of connexin 43 (Cx43)–positive astrocytes in the NAc [129] with the expression of astrocytic GAT3 and EAAT2 in light of the Glu-GABA exchange mechanisms. Also, the induction of EAAT2 expression and trafficking or the motility of the PAPs ([87] and references cited therein) raises the possibility of excitation-induced co-localization of EAAT2 with GAT3 [71, 110, 135, 144, 152]. It is noteworthy that the “gliocentric” (references cited above, and [172]) and “neurocentric” [147] views of inhibitory plasticity corroborate in terms of the chloride gradient shift across the neuronal membrane.

One of the most remarkable manifestations of chloride signaling in the bidirectional communication between neurons and astrocytes in the brain [229] is the spatiotemporal intrinsic optical signal (IOS). The IOS, generated by action potentials and robustly enhanced by disinhibition via GABA_A_ receptor blockade, progresses by activation of Glu receptors and astrocytic Glu transporters [149]. Alteration of tonic inhibition due to EAAT2-mediated Glu-GABA exchange occurs at the astrocytic leaflets preferentially contacting synapses [51] of synaptic islands [63]. These findings also point to the significance of EAAT2 activation–induced astrocytic GAT3 reversal not only in terms of extrasynaptic GABA_A_ receptor activation but also as a mechanism to sensitively modulate chloride gradient and neuronal excitability in this way [165]. From a teleological point of view, MSNs with mixed glutamatergic-GABAergic phenotypes fit the mechanistic clue.

Glu receptor pharmacology may also give an insight into the role of astrocyte activation mechanisms. For example, activation of the group 1 metabotropic Glu receptor (mGluR5) expressed by NAc-resident astrocytes results in a prolonged astrocyte-dependent gliotransmission and stimulation of NMDA receptor–dependent slow inward current in MSNs [41, 46]. In addition to its vital role for promoting resilience to chronic stress [191], accumbal mGluR5s do impact drug-related behaviors. Furthermore, the inhibitory control of astrocyte activation pathways by antagonists of mGluR5 can interfere with cocaine-seeking behavior [111, 204]. Cocaine withdrawal impairs mGluR5-dependent long-term depression in the shell neurons of NAc [77]. It is to note that mGluR1 and mGluR5 modulate distinct excitatory inputs to the NAc shell [212]. The involvement of astrocytic metabotropic Glu receptor is therefore consistent with the positive feedback cell signaling nucleation model of astrocyte dynamics [118].

**Fig. 1:**
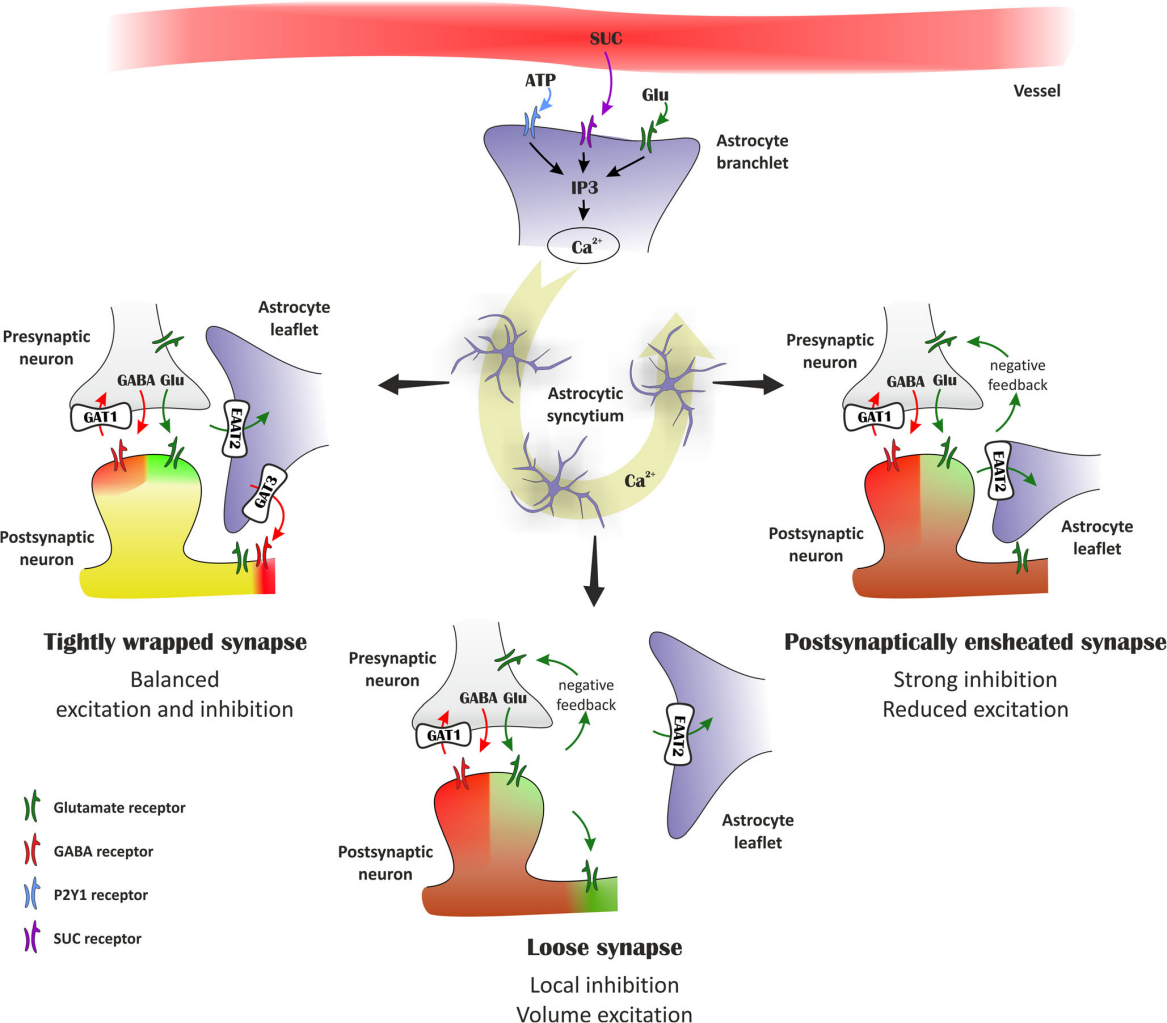
Astrocytes may dynamically control plasticity of mixed inhibitory and excitatory synapses. In the tightly wrapped synapse (left), Glu transporters facing the synapse can quickly take up released Glu, thereby preventing the activation of extrasynaptic Glu receptors. The simultaneous activation of synaptic GABA and Glu receptors results in balanced excitation and inhibition. During intense excitation, EAAT2 activity may also induce GABA release by reverse transport, thereby generating tonic inhibition [68, 69]. When astrocytic leaflets are withdrawn from the synapse (bottom), reduced Glu uptake leads to activation of presynaptic mGluRs inhibiting further Glu release and of extrasynaptic Glu receptors increasing tonic excitation. Asymmetric astrocytic coverage of axonal boutons and dendritic spines (right) [51] favors reduced Glu release by activating presynaptic mGluRs, resulting in a dominantly inhibitory response following GABA/Glu co-release

Further, we can speculate about the significance of the involvement of other G protein–coupled receptors, such as accumbal P2Y1 or Sucn1. Indeed, we can observe slow, recurrent calcium dynamics at a rate of about 3–4 every 10 min evoked by ATP or energy metabolites [129, 131]. Such a recurrent and pacemaker activity of astrocytes has already been described [153, 160, 161] and been related to gliotransmitter (Glu/Asp) release–dependent sustained neuronal activity. By itself, astrocyte activation–related intermittent calcium and sodium transients [[86]; Kirischuk et al., 2017] are consistent with the ideas of (i) flexible tuning of the tripartite synapses, (ii) linked domains of astrocytic syncytium via neuro-glia coupling, and (iii) negative feedback through the astrocytic Glu-GABA exchange signaling. According to this hypothesis (Fig. 1), astrocytes are ideally positioned to control the plasticity of mixed Gluergic/GABAergic synapses. Depending on local activation and propagating Ca^2+^ waves through the astrocytic syncytium due to high-frequency stimulation, cocaine exposure, lactation, or other stimuli, NAc astrocytes can adjust their morphology [155, 205, 206] resulting in different coverages of the synapse. Since Glu uptake and spillover is crucially dependent on astrocytic coverage, the tightness of astrocyte wrapping of the synapse can finely tune the balance between inhibitory and excitatory outcomes (Fig. 1). Indeed, in a rodent model of ethanol self-administration using astrocyte-specific designer receptors to reduce ethanol self-administration, Glu-based bidirectional neuron-astrocyte communication was found in the NAc core, claimed to be important for circuitry guiding motivated behavior [24]. Evidence on interglial gap junction (GJ) channel coupling as a causative agent was also provided [24]. Similarly, activation of an astrocyte-specific designer Gq receptor selectively initiates Glu release and inhibits cue-induced cocaine seeking [182]. Increased neuronal activity and long-term potentiation induce a broader coverage of synapses by PAPs. Strong, prolonged activation, like lactation, results in a decrease in PAP coverage (Fig. 1) [17].

## The Macrosystem NAc—from Motivation to Action

**Fig. 2:**
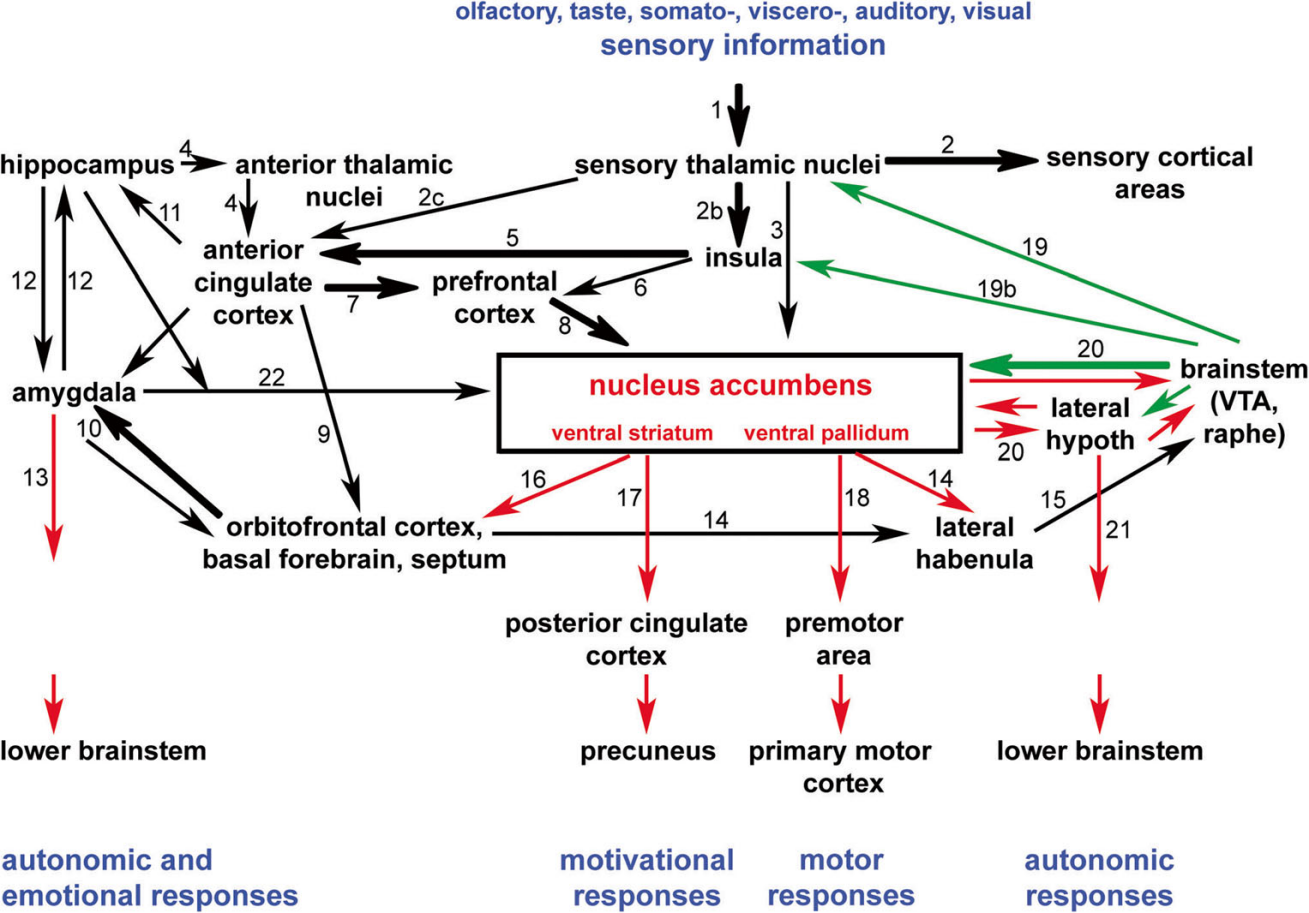
Afferent and efferent connections of the accumbens/ventral striatum. The scheme represents the neuronal pathway interconnectivity converging onto and originating from the nucleus accumbens (NAc)/ventral striatum/pallidum. Different modalities of sensory information reach the NAc through the sensory thalamic nuclei directly and indirectly, too. These glutamatergic inputs (black arrows) are combined in the NAc with monoaminergic (dopaminergic and serotonergic) inputs (green) from the ventral tegmental area (VTA) and the raphe nuclei, respectively. The projections from the NAc are inhibitory (red arrows). Efferent projections to cerebral brain regions initiate motivational and motor responses, while indirect output to the lateral hypothalamus and the amygdala initiates autonomic and emotional responses. Thick arrows represent particularly massive projections. The white matter tracts where the different pathways are located are indicated by numbers as follows: (1) ascending somato- and viscerosensory pathways to the thalamus; (2) thalamocortical radiation; (2b) sensory inputs to the posterior insular cortex; (2c) sensory inputs to the anterior cingulate cortex; (3) thalamo-striatal and pallidal projections; (4) hippocampal-anterior thalamic-anterior cingulate cortex connections through the “Papez circle”; (5) anterior insular projections to the anterior cingulate cortex (the two hubs of the salience network); (6) direct anterior insular projections to the prefrontal cortex (to the ventrolateral and dorsolateral prefrontal cortex); (7) bilateral anterior cingular-prefrontal cortical fiber connections; (8) prefrontal neuronal feedback to the nucleus accumbens; (9) bilateral anterior cingular-orbitofrontal cortical fiber connections; (10) bilateral connections between the orbitofrontal cortex and the amygdala (uncinate fascicle); (11) anterior cingulate projections to the hippocampus through the parahippocampal cortex; (12) amygdala connections with the hippocampus (via peri- and entorhinal cortex); (13) descending amygdala projections to the lower brainstem (partly through the stria terminalis); (14) stria medullaris thalami; (15) fasciculus retroflexus; (16) nucleus accumbens, ventral striatal/pallidal projections to the orbitofrontal cortex, basal forebrain, and septum; (17) fibers from the nucleus accumbens/ventral striatal and pallidal neurons in the fronto-parietal neuronal connections (“dorsal default mode network”); (18) nucleus accumbens, ventral striatal/pallidal projections to the premotor and motor cortical areas; (19) ascending brainstem dopaminergic (from the ventral tegmental area) and serotinergic fibers (from the midline midbrain raphe nuclei) to the thalamus (one portion of the ascending reticular activating system); (19b) ascending brainstem dopaminergic and serotinergic fibers to the insula; (20) medial forebrain bundle; (21) descending fibers from the lateral hypothalamus to the lower brainstem; (22) ventral amygdalofugal pathway

Macrosystem (Fig. 2) metaplasticity may possibly be better characterized as an arousal-driven specific acquisition/approach and reinforcement of accumbal circuitries, rather than as a general adaptive NAc response [22, 23, 57, 61, 82, 127, 136, 154, 176, 230, 238, 240]. The NAc is embedded into the larger cortico-basal ganglia-thalamo-cortical loop and is considered to be a main integration center of the basal ganglia. Being part of the ventral striatum, the major connection to the NAc is from afferents of pyramidal cells populating the layers II–VI of parts of the prefrontal cortex, with an indirect massive contribution from the anterior cingulate cortex. These afferents supply the higher order perceptive, homeostatic, anticipative, and emotional state information to the NAc and represent a major route of sensory information toward the NAc together with some direct thalamic inputs. In addition, a powerful glutamatergic pathway from the basolateral amygdala innervates both the shell and core of the NAc that are implicated in motivational salience, affective behavior, and emotion. The ventral pallidum is the primary output area of NAc. Efferent projections from the NAc are ultimately cortical areas, such as the precuneus via the posterior cingulate cortex and the motor cortex to provide incentives for the execution of motor responses. The NAc can also affect autonomic and emotional responses via the amygdala and the lateral hypothalamus. Importantly, the mesolimbic dopamine (DA) pathway partially via the lateral hypothalamus is responsible for positive reinforcement by reward that can be traced to NAc and ventral pallidum. The more medial areas of the hypothalamus send aversive signals to the same receivers; thus, high spatial and functional selectivity must exist among the adjacent mesolimbic DA fibers. The NAc also receives potent serotonergic inputs that bind to several types of 5HT receptors (5HT1–4). Serotonin potently interferes with the mesolimbic DA pathway and overrides the inhibitory action of DA in selected neurons in the shell region. The NAc can also affect these brainstem centers directly and also indirectly via the orbitofrontal cortex, the basal ganglia, the septum, and the lateral habenula. Several lines of evidence can be found in the realm of expert’s practice and relevant scientific literature. For example, the NAc/ventral striatum of the accumbal macrosystem drives and reinforces reward-associated feeding and sexual or somatic and visceral (loco)motor actions, including repetitive behavior [20, 32, 35, 42, 43, 55, 66, 73, 90, 98, 99, 106, 117, 173, 214, 232, 244]. Motivations, shaping the emotional [76] or cognitive addiction behavior [81, 83, 190, 196] via activating the NAc-hippocampus and hypothalamus-NAc circuits, respectively, have the potential of alternative execution as well. Evidently, the various higher order brain functions, like the motivational, adaptive, and goal-directed behaviors impinging upon and originating from the NAc, underlie why this basal ganglia nucleus function is prone to be hijacked by illicit substances and neurotransmitter mimetics in an abusive manner.

## Conclusions

Future research is needed to unravel the context in which astrocyte activation may specifically stimulate neuronal ensembles of the accumbal macrosystem to function in different directions [26, 58, 115, 116]. Although accumbal circuit connections and silent synapses grant a high degree of both specificity and neuronal metaplasticity potential [108, 109, 151], there seems to remain room for including additional concepts, such as astrocytic “randomness” arising from activity-dependent rearrangements of energy metabolism, morphology, GJ-coupled domains, distal astrocytic processes, or synaptic leaflets during later stages of reinforcement. These studies may also imply that astrocytes do not only act in response to accumbal neuronal ensembles but also combine metabolic energy, they modulate signaling by supporting different (positive and negative) outcomes. Beyond the potential significance of astrocytic interleukin-6 [113] and leptin [134, 224] signaling, or ionotropic/metabotropic Glu receptor subunit-specific synaptic potentiation [3, 75, 108–110, 126], the enhanced allocation of reward-associated gamma oscillations [85] may open up novel therapeutic opportunities.

Below the thunders of the upper deep,

Far far beneath in the abysmal sea,

His ancient, dreamless, uninvaded slee

The Kraken sleepeth:

Tennyson, Alfred Lord: The Kraken

http://www.poemhunter.com/poem/the-kraken-2/

## Abbreviations

5HT: Serotonin (5-hydroxytryptamine)
Ach: Acetylcholine
AKR7A2: Astrocytic succinic semialdehyde/aldo-keto reductase enzyme
AKR1B10: Succinic semialdehyde/aldo-keto enzyme
Akr1b10: Succinic semialdehyde/aldo-keto reductase gene
Aldh5A1: Mitochondrial succinic semialdehyde/aldo-keto reductase enzyme
Asp: Aspartate
BBB: Blood-brain barrier
CEC: Citrate energy cycle
Cx43: Connexin 43
EAAT2: GLT-1, SLC1A2—glial Na^+^ and H^+^ ion-dependent excitatory amino acid transporter type 2 with (1Glu:3Na^+^:1H^+^)_in_/(K^+^)_out_ stoichiometry
DA: Dopamine (3,4-dihydroxyphenethylamine)
FC: Frontal cortex
GABA: γ-Aminobutyric acid
GAD: Glutamic acid decarboxylase
GAT3: SLC6A11—glial Na^+^ and Cl^−^ ion-dependent GABA transporter type 3 with (1GABA:2Na^+^:1Cl^−^)_in_ stoichiometry
GFAP: Glial fibrillary acidic protein
GHB: γ-Hydroxy butyric acid
GJ: Gap junction
Glu: Glutamate
GluA2: AMPA receptor—alpha-amino-3-hydroxy-5-methyl-4-isoxazole propionate receptor
IOS: Intrinsic optical signal
Meth: Methamphetamine, N-methyl-amphetamine
mGluR5: Group 1 metabotropic Glu receptor
NAc: Nucleus accumbens
NMDA: *N*-methyl-*D*-aspartate
Nqo2: NAD(P)H dehydrogenase, quinone 2 gene
PAPs: Perisynaptic astrocytic processes
PV: Parvalbumin
Pvalb: Parvalbumin gene
Sgms2: Sphingomyelin synthase gene
SOM: Somatostatin
SSA: Succinic semialdehyde
SSADH: Succinic semialdehyde dehydrogenase enzyme
Succinate: Sucn
Sucnr1: Succinate receptor 1 gene
VTA: Ventral tegmental area
